# Comparison of efficacy between endoscope-assisted anterior cervical discectomy and fusion (ACDF) and open ACDF in the treatment of single-segment cervical spondylotic myelopathy

**DOI:** 10.1186/s13018-023-04514-w

**Published:** 2024-01-05

**Authors:** Zhi-Peng Wu, Zhao-yong Wei, Xiao-Lei Song

**Affiliations:** Department of Minimally Invasive Spinal Surgery, Angang General Hospital, Middle Section of Gangsan Road, Yindu District, Anyang, 445000 China

**Keywords:** Anterior cervical discectomy and fusion, Cervical spondylotic myelopathy, Clinical efficacy, Intervertebral bone graft fusion, Spinal endoscopy, Zero-plant

## Abstract

**Background:**

In this study, we compared the clinical efficacy of endoscope-assisted anterior cervical discectomy and fusion (ACDF) with open ACDF in the treatment of single-segment cervical spondylotic myelopathy.

**Methods:**

A retrospective analysis was performed on 52 patients with single-segment cervical spondylotic myelopathy between June 2021 and February 2022, including 33 males and 19 females, with a mean age of 58.42 ± 9.26) years. Among them, 28 patients were treated with endoscope-assisted ACDF (Group A), including 2 cases of C4/5 segment, 16 cases of C5/6 segment, and 10 cases of C6/7 segment; 24 patients were treated with open ACDF (Group B), including 4 cases of C4/5 segment, 11 cases of C5/6 segment, and 9 cases of C6/7 segment. The operation time, intraoperative blood loss, hospital stay, and complications were recorded and compared between the two groups. The Visual Analogue Scale (VAS) and the Japanese Orthopaedic Association (JOA) score were used for clinical evaluation during the follow-up in the 1st month and 3rd month after surgery, and at the final follow-up.

**Results:**

The 52 patients were followed up on average for 13.04 months (12–17 months). The operation time in Group A and Group B was (105.18 + 8.66) minutes and (81.88 + 6.05) minutes, the intraoperative blood loss was (84.29 + 13.45) mL and (112.92 + 17.81) mL, and the hospital stay was (6.75 + 1.29) days and (7.63 + 1.41) days, respectively. The difference between the two groups was statistically significant (*P* < 0.05). The VAS and JOA scores in the 1st month and the 3rd month after surgery and the last follow-up significantly improved in both groups compared with those before surgery (*P* < 0.05). The VAS and JOA scores of Group A in the 1st month, 3rd month after surgery, and the last follow-up were better than those in Group B (*P* < 0.05). The complication rate in Group A was 7% (2/28), which was not significantly different from the 17% (4/24) in Group B (*P* > 0.05).

**Conclusion:**

Both endoscope-assisted ACDF and open ACDF can achieve satisfactory clinical efficacy in the treatment of single-segment cervical spondylotic myelopathy. Although the operation time of endoscope-assisted ACDF is prolonged, it has the advantages of clear vision, thorough decompression, less blood loss, and reduced risk of nerve damage, and is worthy of clinical promotion and application.

## Background

Cervical spondylotic myelopathy (CSM) is caused by chronic mechanical compression from the ventral and dorsal sides of the spinal cord, causing spinal cord ischaemia and parenchymal pathological changes in the spinal cord, which in turn leads to spinal cord dysfunction. Surgical intervention is often required in cases where nonsurgical treatment is ineffective or spinal cord dysfunction is progressively worsening [1, 2]. Anterior cervical discectomy and fusion (ACDF) is the "gold standard" for the treatment of CSM, as it can directly relieve the spinal cord and nerve compression and alleviate patient symptoms. However, there are problems such as a narrow surgical field, poor coordination between the surgeon and assistants, difficulty in stopping bleeding, and incomplete decompression [3, 4]. The use of spinal endoscopy has made minimally invasive cervical spine surgery a possibility thanks to advancements in the field of minimally invasive spine surgery [5–7]. In this study, we compared the efficacy of endoscope-assisted ACDF with open ACDF in the treatment of single-segment CSM and explored the advantages of spinal endoscopy in ACDF.

## Data and methods

### General clinical data

A cohort of 52 patients with single-segment CSM who visited our hospital between June 2021 and February 2022 were included in this study as per the inclusion and exclusion criteria. All surgeries were performed by the same chief physician. There were 33 males and 19 females, with a mean age of 58.42 ± 9.26 years. Among them, 28 patients were treated with endoscope-assisted ACDF (Group A), which included 17 males and 11 females, aged between 43 to 75 years, with a mean age of 60.39 ± 9.57 years; surgical segments: 2 cases of C4/5 segment, 16 cases of C5/6 segment, 10 cases of C6/7 segment. There were 24 patients treated with open ACDF (Group B), which included 16 males and 8 females, aged between 42 and 77 years, with an average age of 56.13 ± 8.49 years; surgical segments: 4 cases of C4/5 segment, 11 cases of C5/6 segment, 9 cases of C6/7 segment. All patients displayed the symptoms and signs of typical CSM, such as sensory disturbances in the extremities, inability to hold objects, feeling of stepping on cotton in both feet, active or hyperactive tendon reflexes in the extremities, and positive pathological signs. Preoperative routine examinations included cervical spine frontal, lateral and dynamic X-ray films, cervical spine computed tomography (CT) and magnetic resonance imaging (MRI), to confirm the diagnosis. The preoperative symptoms, signs, and imaging findings were consistent. No significant difference was found in gender, age, and surgical segment between the two groups (*P* > 0.05), and they were comparable (Table 1).

**Table 1:** Comparison of the baseline data between groups

Items	Group A (n = 28)	Group B (n = 24))	*X*^2^/t value	*P* value
Gender (n)			0.02	0.88
Man	17	16		
Female	11	8		
Age (X + s, year)	60.39 + 9.57	56.13 + 8.49	1.69	0.26
Operative segment			1.35	0.51
C4/5	2	4		
C5/6	16	11		
C6/7	10	9		

### Inclusion and exclusion criteria

#### Inclusion criteria

1. Symptoms, signs, and imaging examinations are consistent with the diagnosis of single-segment CSM. 2. The affected segment is consistent with the clinical manifestations, and imaging shows degenerative changes in the cervical spine, such as intervertebral disc herniation, osteophyte hyperplasia at the posterior edge of the vertebral body, and other pathological changes. 3. Patients who fail conservative treatment or have progressive worsening of spinal cord nerve function, which requires surgical intervention. 4. Patients who have no contraindications related to surgery. 5. Patients and their families gave informed consent and are willing to accept the surgical treatment.

#### Exclusion criteria

1. Patients with two-segment and multi-segment CSM. 2. Patients with cervical spinal cord injury and compression caused by congenital cervical spinal stenosis, hypertrophy of ligament flavum, and ossification of the posterior longitudinal ligament. 3. Patients with severe organic diseases and coagulation dysfunction who cannot tolerate surgery. 4. Patients with infection, tumour, fracture, and severe osteoporosis. 5. Patients who have mental disorders and cannot cooperate till the completion of this study.

### Surgical method

Surgical Approach to the Anterior Cervical Spine: Patients were positioned supine on a fluoroscopic operating table and placed under general anaesthesia with endotracheal intubation. A transverse incision, typically measuring 4–5 cm in length, was meticulously made on the right side of the neck. Subsequently, the surgical field was exposed through the layers of skin, subcutaneous tissue, and platysma. Following the platysma incision, the sternocleidomastoid muscle and strap muscles became visible. Further dissection revealed the natural fissure between the carotid sheath (located deep to the sternocleidomastoid muscle) and the tracheoesophageal sheath (positioned deep to the strap muscles), which was employed as the surgical corridor. A gradual dissection approach was then adopted to access the anterior aspect of the cervical spine.

Subsequently, a positioning needle was meticulously inserted, and the surgical segment's precise location was verified using a C-arm X-ray machine. The bilateral longus colli muscles were dissected, extending from the lateral subperiosteal space to the anterior surface of the bilateral uncovertebral joints, to facilitate enhanced exposure. Spinal nails were meticulously placed in the upper and lower vertebral bodies adjacent to the targeted surgical segment, and a Caspar cervical retractor was employed to secure and moderately expand the intervertebral space. Under direct visualization, a scalpel was used to incise the anterior fibrous ring, followed by the use of rongeurs and curettes to excise approximately 2/3–3/4 of the anterior fibrous ring and the nucleus pulposus, extending to both sides of the uncovertebral joints.

#### Surgical method for group A

**Fig. 1:**
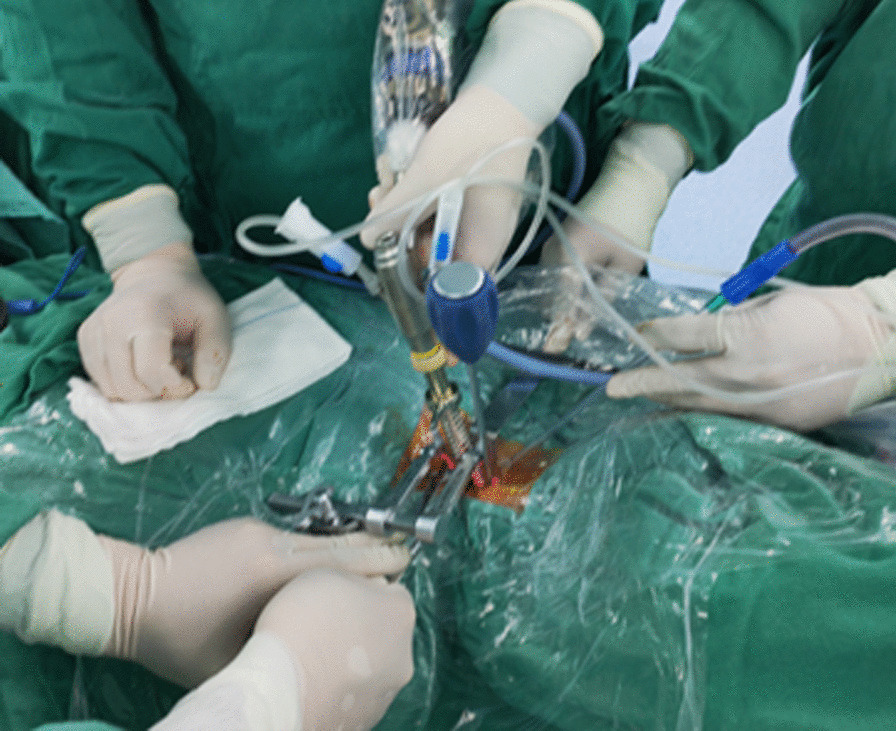
Intraoperative imaging: After determining the C5/6 segment through the traditional open approach, the spinal endoscopic system was placed

**Fig. 2:**
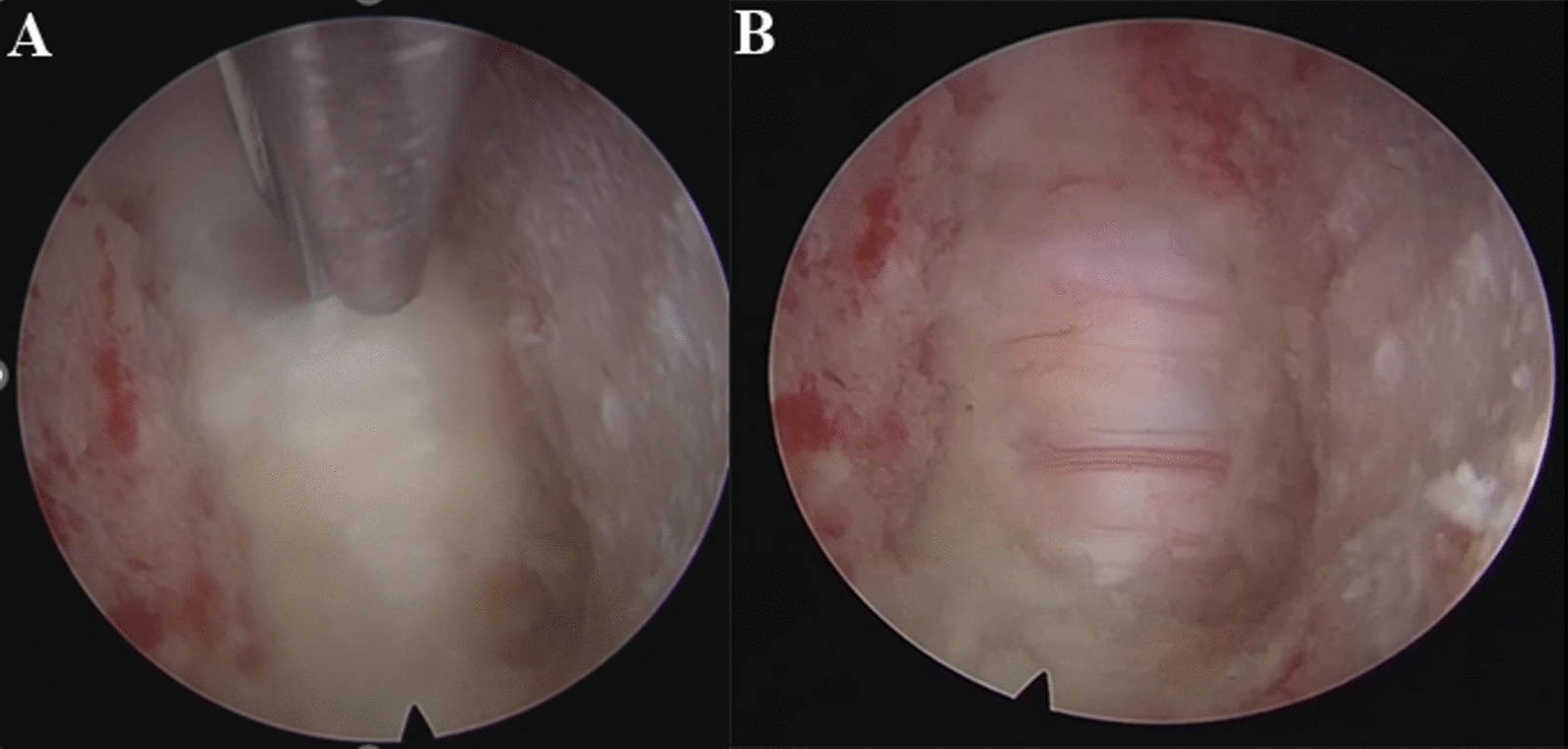
Intraoperative microscopic figures: **A** Laminar forceps were employed to meticulously grasp and excise the intervertebral disc, posterior longitudinal ligament, and hyperplastic osteophytes, all conducted under the direct guidance of endoscopic visualization. **B** The dural sac was fully exposed following the decompression procedure conducted under endoscopic guidance

The large channel spinal endoscopic system (Joimax, Germany) was used for Group A. Under direct endoscopic vision, the remaining intervertebral disc at 1/3–1/4 of the posterior vertebral body was cleaned with an endoscopic spatula and laminectomy rongeur (Fig. 1). The cartilage endplate and the hyperplastic osteophytes at the posterior edge of the intervertebral space were removed with a microscopic high-speed abrasion drill. After thinning the hyperplastic osteophytes, the remaining osteophytes at the posterior edge of the intervertebral space were removed with a microscopic cervical laminectomy rongeur, till the bilateral uncovertebral joints. A "trapezoid" operating space at the back of the vertebral body was expanded and established. The posterior fibrous ring and posterior longitudinal ligament were cut open with a microscopic cervical laminectomy rongeur (Fig. 2A). The dural sac was exposed and the possibility of nucleus pulposus residue was explored before thorough decompression (Fig. 2B). After complete haemostasis using radiofrequency, the large channel spinal endoscope was withdrawn after confirming that the pulsation of the dural sac was good, and there was no tortuous venous plexus on the surface of the spinal cord. An appropriate zero-profile cage was selected, and the autologous bone was implanted inside the cage and placed in the responsible intervertebral space. Then, two locking screws were fastened onto the vertebral body. C-arm X-ray fluoroscopy was performed to visualize the positioning of the cage and screws. Subsequently, an indwelling drainage tube was placed, the wound was sutured layer by layer, and sterile dressing was used.

#### Surgical method for group B

The traditional open ACDF procedure was adopted in Group B. Under direct vision, the remaining intervertebral disc at 1/3–1/4 of the posterior vertebral body was cleaned with a spatula and laminectomy rongeur. The hyperplastic osteophytes at the posterior edge of the intervertebral space were removed with a laminectomy rongeur till the bilateral uncovertebral joints. The operating space at the back of the vertebral body was established. The posterior fibrous ring and posterior longitudinal ligament were cut open with a cervical laminectomy rongeur. The dural sac was exposed and the possibility of nucleus pulposus residue was explored before thorough decompression. After complete haemostasis using radiofrequency, it was confirmed that the pulsation of the dural sac was good, and there was no tortuous venous plexus on the surface of the spinal cord. An appropriate zero-plant cage was selected, and the autologous bone was implanted inside the cage and placed in the appropriate intervertebral space. Then, two locking screws were fastened onto the vertebral body. C-arm X-ray fluoroscopy was performed to visualize the positioning of the cage and screws. Subsequently, an indwelling drainage tube was placed, the wound was sutured layer by layer, and sterile dressing was used.

### Postoperative management

After the operation, mannitol and antibiotics were routinely administered by intravenous drip for 3 days, and the drainage tube was removed 48 h after the operation. Patients were made to wear the neck brace when walking. Conventional oral administration of mecobalamin was administered to promote the recovery of nerve function, and the stitches were removed 5–7 days after surgery. Patients were instructed to wear the neck brace for a further 8 weeks.

### Observation indicators and evaluation methods

① Comparison of general data of patients in the two groups: age, gender, surgical segment, operation time, intraoperative blood loss, hospital stay and follow-up duration of patients in the two groups were compared. ② Comparison of clinical scores of patients in the two groups: the visual analogue scale (VAS), Japanese Orthopaedic Association (JOA) score, and JOA improvement rates before surgery, in the 1st month after surgery, 3rd month after surgery, and at the final follow-up of the two groups were compared. The VAS scores ranged from 0 to 10 points, with lower scores indicating less pain. The JOA scores ranged from 0 to 17 points, with higher scores indicating better neurological recovery. JOA improvement rate (%) = (postoperative JOA score—preoperative JOA score)/(17—preoperative JOA score) × 100%. ③ Comparison of complications between the two groups: The occurrence of postoperative complications in the two groups was recorded and compared.

### Statistical methods

SPSS 22.0 statistical software was adopted. The measurement data are described as mean ± standard deviation, and the counting data are described as percentage. The ages, VAS scores, JOA scores, JOA improvement rates, operation time, intraoperative blood loss, and follow-up duration of patients in the two groups were compared using an independent sample *t*-test, and gender, surgical segment, and complications were compared using a the chi-squared test. *P* < 0.05 indicated a statistically significant difference.

## Results

Comparison of general data of the two groups: No significant difference was found in the general data including gender, age, and surgical segment between the two groups (*P* > 0.05) (Table 1).

All patients were followed up for 12 to 17 months, with an average duration of (13.04 ± 1.39) months. Comparison of operation time, intraoperative blood loss and hospital stay between the two groups: The operation time of patients in Group A was (105.18 + 8.66) minutes, and the difference was statistically significant compared to the (81.88 + 6.05) minutes in Group B (*P* < 0.05). The intraoperative blood loss of patients in Group A was (84.29 + 13.45) mL, and the difference was statistically significant compared to the (112.92 + 17.81) mL in Group B (*P* < 0.05). The length of hospital stay was (6.75 + 1.29) days in Group A, and the difference was statistically significant compared to the (7.63 + 1.41) days in Group B (*P* < 0.05) (Table 2).

**Table 2:** Comparison of the operation time, intraoperative blood loss, length of stay and follow-up time between groups

Items	Group A (n = 28)	Group B (n = 24))	*X*^2^/t value	*P* value
Operation time (X + s, min)	105.18 + 8.66	81.88 + 6.05	11.07	0.00
Intraoperative blood loss (X + s, ml)	84.29 + 13.45	112.92 + 17.81	− 6.59	0.00
Length of hospital stay (X + s, d)	6.75 + 1.29	7.63 + 1.41	− 2.33	0.02
Follow-up time (X + s, month)	12.71 + 0.94	13.42 + 1.72	− 1.76	0.07

Comparison of VAS and JOA scores at various time points before and after surgery between the two groups: There was no significant difference in the preoperative VAS and JOA scores between the two groups (*P* > 0.05). The VAS and JOA scores in the 1st month after surgery, 3rd month after surgery, and at the final follow-up were significantly lower than those before surgery in the two groups (*P* < 0.05). There was a statistically significant difference in the VAS scores, JOA scores, and JOA improvement rates in the 1st month after surgery, 3rd month after surgery, and at the final follow-up between the two groups (*P* < 0.05) (Table 3).

**Table 3:** Comparison of the VAS, JOA score, and JOA recovery rate at pre-operation and each time point postoperatively and between groups

Time	VAS scores		JOA scores		JOA improvement rates	
	Group A	Group B	Group A	Group B	Group A	Group B
Preoperative	5.36 + 1.97	5.21 + 1.69	7.63 + 1.47	8.25 + 1.51		
*Postoperation*						
1st month after surgery	2.82 + 0.72^a,b^	3.38 + 0.97^b^	13.35 + 1.16^a,b^	12.46 + 1.67^b^	60.32 + 14.00^a^	48.30 + 18.82
3rd month after surgery	2.15 + 0.65^a,b^	2.58 + 0.78^b^	14.32 + 0.98^a,b^	13.29 + 1.30^b^	70.76 + 12.29^a^	57.93 + 13.67
Final follow-up	1.21 + 0.42^a,b^	1.54 + 0.58^b^	14.61 + 0.83^a,b^	13.96 + 0.95^b^	73.81 + 10.48^a^	65.59 + 9.31

^a^Compared with group B, *P* < 0.05; ^b^Compared with preoperative, *P* < 0.05

Comparison of the complications between the two groups: The main postoperative complications that ensued in the two groups were 1 case (2%) of prevertebral subcutaneous haematoma, 3 cases of dysphagia (6%), and 2 cases of hoarseness (4%). Of those, the complication rate in Group A was 7% (2/28), including 1 case of dysphagia and 1 case of hoarseness. The complication rate in Group B was 17% (4/24), including 1 case of prevertebral subcutaneous haematoma, 2 cases of dysphagia, and 1 case of hoarseness. The two groups did not have complications such as cerebrospinal fluid leakage, postoperative infection, and loose fusion cage. Also, no significant difference was found in the complications between the two groups (*P* > 0.05).

## Discussion

CSM is often secondary to pathological changes such as disc herniation, osteophytes at the posterior edge of the vertebral body, hypertrophy of ligament flavum, and ossification of the posterior longitudinal ligament, which cause chronic mechanical compression of the spinal cord, in turn inducing clinical manifestations related to spinal cord dysfunction [8, 9]. Surgical intervention is indicated if nonsurgical treatment is ineffective or spinal cord dysfunction aggravates progressively [2, 10]. The principle of anterior approach surgery is to relieve spinal cord compression factors, restore spinal stability, maintain intervertebral height and physiological lordosis, and recover spinal cord function [11, 12]. Open ACDF is the standard procedure for the treatment of CSM/Cervical Spondylotic Radiculopathy (CSRR) secondary to soft disc herniation at the disc level or osteophyte hyperplasia at the posterior edge of the vertebral body, with satisfactory clinical efficacy [4, 13–15]. However, the surgical field in this surgical method is narrow, haemostasis is difficult, anatomical structures such as the posterior longitudinal ligament and dural sac are difficult to identify, and there is a risk of spinal nerve injury [14–16].

With the development of minimally invasive spinal surgery techniques, micro-endoscopic techniques have been gradually applied to cervical ACDF surgery. It has the advantages of enlarging the surgical field, improving the brightness of the surgical field, making the surgical field clearer, making the surgical anatomical separation process more accurate and meticulous, and reducing the risk of spinal cord nerve injury [6, 14, 15]. However, micro-endoscopy has drawbacks such as poor hand–eye coordination, inability to maintain focus on the surgical plane, incorrect instrument placement that interferes with the surgical field, and neck and shoulder strain for the surgeon [5, 17]. Considering these shortcomings of micro-endoscopy, we tried using large channel spinal endoscopy instead of micro-endoscopy to perform ACDF surgery, in order to provide clinical guidance for spinal endoscopy in the treatment of cervical spondylosis.

At present, the application of spinal endoscopic technology in the treatment of cervical degenerative diseases is becoming perceptibly more sophisticated, and the indications have gradually expanded from early cervical spondylotic radiculopathy to CSM [7, 18, 19]. Anterior cervical approach spinal endoscopic techniques include transdisc and transvertebral approaches for the removal of ventral compression factors from spinal nerve roots [19–21]. However, no clinical study has adopted spinal large channel endoscopy in the treatment of CSM with anterior cervical ACDF technology. Compared with the traditional open ACDF group, the endoscope-assisted ACDF group had a longer operation time. The main reasons are as follows: 1. Installation of spinal endoscopic instruments. 2. The surgeon performed this technique for the first time, and needed to gradually get familiar with microscopic anatomy and surgical operation, which has a learning curve. 3. The microscopic operation is more accurate and meticulous, the instrument model is small and thin, and it takes a longer time to complete thorough decompression of bilateral uncovertebral joints, posterior longitudinal ligaments, and the dural sac. Compared with the traditional open ACDF group, intraoperative blood loss was significantly reduced in the endoscope-assisted ACDF group. Spinal endoscopy can magnify the surgical field by a factor of 10 while obtaining a clear surgical field by continuous irrigation with water, which is conducive to quickly identifying bleeding points [22]. Also, the application of microscopic radiofrequency and bone wax can effectively stop bleeding. The VAS scores and JOA scores in the 1st month and 3rd month after the surgery and at the final follow-up in the two groups had significantly improved as compared with that before surgery (*P* < 0.05). Moreover, the VAS scores, JOA scores, and JOA improvement rates in the 1st month and 3rd month after surgery and at the final follow-up in the endoscope-assisted ACDF group were significantly better than those in the traditional open ACDF group. This is primarily related to the thorough decompression of spinal nerve roots, mainly the complete decompression of posterior longitudinal ligaments and dural sac, extraction of the herniated intervertebral disc, and removal of hyperplastic osteophytes at the posterior edge of the vertebral body and 1/2 of bilateral uncovertebral joints. The postoperative complications in the two groups mainly included 1 case (2%) of prevertebral subcutaneous haematoma, 3 cases (6%) of dysphagia, and 2 cases (4%) of hoarseness. Among them, one case of dysphagia and one case of hoarseness occurred in the endoscope-assisted ACDF group. There was no significant difference in the complications between the two groups (*P* > 0.05). The clinical results suggest that spinal endoscopic techniques can enable safe and effective ACDF surgery without increasing the surgical risk. They also apparently reduce the risk of nerve root dural sac injury.

To solve the shortcomings of microscopy and traditional open surgery, we organically combined spinal endoscopy with ACDF techniques. After clinical practice, it has been found that endoscope-assisted ACDF has the following advantages: 1. The spinal large channel endoscope is placed on the surface of the affected intervertebral space, and the light source can effectively illuminate the surgical area. The surgical field can be magnified 10x, a clear surgical field can be obtained by continuous irrigation with water, and the bleeding can be effectively stopped with microscopic radiofrequency and bone wax. 2. The spinal large channel endoscope is a coaxial operating system that conforms to the operating habits of endoscopists while avoiding mutual interference between instruments and improving surgical efficiency. 3. The large spinal endoscopic system can upload the surgical field pictures to the display, which can reduce the incidence of degenerative diseases of the cervical spine and shoulder joints caused by long-term neck flexion in surgeons [6, 17]. 4. The anatomical structure is clearly identified, which reduces the risk of injury. Under direct vision, the posterior longitudinal ligament and dural sac can be completely decompressed, the herniated intervertebral disc and the residual cartilage endplate can be extracted, and the hyperplastic osteophytes at the posterior edge of the vertebral body and 1/2 of the bilateral uncovertebral joints can be removed. 5. The outer cannula of the spinal large channel is placed on the surface of the intervertebral space, and the endoscope and outer cannula do not sink into the intervertebral space and cause spinal nerve injury. At the same time, the spinal instruments are small and thin, easy to operate, and stable to hold, which can reduce operation-related injuries. 6. The spinal endoscope is flexible, and there is no need to focus repeatedly while adjusting the lens, which can increase the surgical operation range, improve surgical efficiency, and reduce the surgical blind area. This study has shortcomings such as a small sample size and short follow-up time, and further verification by large-sample, multi-centre, randomized controlled trials is needed in the future.

## Conclusion

To summarize, the results of this study showed that both endoscope-assisted ACDF and open ACDF can achieve satisfactory clinical efficacy in the treatment of single-segment CSM. Although the operation time of endoscope-assisted ACDF surgery is prolonged, spinal endoscopic surgery provides a clearer field of view, with less surgical injury, more delicate operation, and less intraoperative bleeding, which can reduce the potential risk of nerve damage.

## Data Availability

The datasets used and/or analysed during the current study are available from the corresponding author upon reasonable request.

## Abbreviations

Endoscope-assisted ACDF: Endoscope-assisted anterior cervical discectomy and fusion
VAS: Visual analogue scale
JOA: Japanese orthopaedic association
CSM: Cervical spondylotic myelopathy
CT: Computer tomography
MRI: Magnetic resonance imaging
